# Early detection of *Pseudomonas aeruginosa *– comparison of conventional versus molecular (PCR) detection directly from adult patients with cystic fibrosis (CF)

**DOI:** 10.1186/1476-0711-3-21

**Published:** 2004-10-20

**Authors:** Jiru Xu, John E Moore, Philip G Murphy, B Cherie Millar, J Stuart Elborn

**Affiliations:** 1 Northern Ireland Public Health Laboratory, Department of Bacteriology, Belfast City Hospital, Lisburn Road, Belfast, Northern Ireland, BT9 7AD, UK; 2 School of Biomedical Sciences, University of Ulster, Cromore Road, Coleraine, Co. Londonderry, Northern Ireland, BT52 1SA, UK; 3 Northern Ireland Regional Adult Cystic Fibrosis Centre, Level 8, Belfast City Hospital, Lisburn Road, Belfast, Northern Ireland, BT9 7AB, UK; 4 Department of Respiratory Medicine, The Queen's University of Belfast, Belfast City Hospital, Lisburn Road, Belfast, Northern Ireland, BT9 7AB, UK

## Abstract

**Background:**

*Pseudomonas aeruginosa *(PA) is the most important bacterial pathogen in patients with cystic fibrosis (CF) patients. Currently, routine bacteriological culture on selective/non- selective culture media is the cornerstone of microbiological detection. The aim of this study was to compare isolation rates of PA by conventional culture and molecular (PCR) detection directly from sputum.

**Methods:**

Adult patients (n = 57) attending the regional adult CF centre in Northern Ireland, provided fresh sputum following airways clearance exercise. Following processing of the specimen with sputasol (1:1 vol), the specimen was examined for the presence of PA by plating onto a combination of culture media (*Pseudomonas *isolation agar, Blood agar & McConkey agar). In addition, from the same specimen, genomic bacterial DNA was extracted (1 ml) and was amplified employing two sequence-specific targets, namely (i) the outer membrane protein (*opr*L) gene locus and (ii) the exotoxin A (ETA) gene locus.

**Results:**

By sputum culture, there were 30 patients positive for PA, whereas by molecular techniques, there were 35 positive patients. In 39 patients (22 PA +ve & 17 PA -ve), there was complete agreement between molecular and conventional detection and with both PCR gene loci. The *opr*L locus was more sensitive than the ETA locus, as the former was positive in 10 more patients and there were no patients where the ETA was positive and the *opr*L target negative. Where a PCR +ve/culture -ve result was recorded (10 patients), we followed these patients and recorded that 5 of these patients converted to being culture-positive at times ranging from 4–17 months later, with a mean lag time of 4.5 months.

**Conclusions:**

This study indicates that molecular detection of PA in sputum employing the *opr*L gene target, is a useful technique in the early detection of PA, gaining on average 4.5 months over conventional culture. It now remains to be established whether aggressive antibiotic intervention at this earlier stage, based on PCR detection, has any significant benefits on clinical outcome.

## Introduction

Cystic fibrosis [CF] is the most commonly inherited fatal disease in persons originating from a white and European background, currently affecting approximately 30,000 adults and children in the US [1]. The defective gene carrying the mutation is carried in one in every 31 Americans [one in 28 Caucasians], equating to more than 10 million people being a symptomless carrier of the defective gene [1]. It is an autosomal recessive condition whereby two alleles carrying a polymorphism in the cystic fibrosis transmembrane conductance regulator [CFTR] gene phenotypically manifest the disease state through a variety of multiorgan problems, associated with a pharmacological disfunction to regulate sodium and chloride secretion across cell membranes. The most common complication of CF is the recurrence of chronic chest infections usually caused by bacterial pathogens [2]. CF patients continue to suffer from recurrent and chronic respiratory tract infections and most of their morbidity and mortality is due to such infections throughout their life [3]. These infections are usually dominated by Gram-negative organisms, especially by the pseudomonads, including *Pseudomonas aeruginosa*, *Burkholderia cepacia *and *Stenotrophomonas maltophilia*. However, with modern antibiotic management with improved antimicrobial agents, such as the aminoglycosides and carbapenems, CF patients have an improved survival, resulting in more adults in employment.

Previous studies have described alternative laboratory markers to conventional bacteriological culture for the detection of *Pseudomonas aeruginosa *in CF patients.[4,5] These markers have included serological testing of CF patients' sera for the presence of antibody to *P. aeruginosa*,[4] as well as molecular detection of DNA signature sequences of *P. aeruginosa *in CF patients [5]. Presently, the routine employment of molecular (PCR) detection of *P. aeruginosa *directly from the sputum of CF patients in the UK and Ireland is uncommon. Most clinical microbiology laboratories in the UK and Ireland, which support a CF centre, have developed standard operating procedures (SOPs) for the isolation of this organism from patients' sputum. Hence, it was the aim of this study to compare the conventional and molecular detection of *P. aeruginosa *from the sputum of adult patients attending the adult centre in Northern Ireland, as well as to estimate the lag time of conventional culture to detection, compared with molecular (PCR) detection.

## Materials & Methods

### Qualitative conventional detection of *Pseudomonas aeruginosa *from sputum

Duplicate sputa (1 ml minimum) specimens were collected from 57 adult patients with a well characterized history of CF in sterile (100 ml) plastic disposable containers. Sputum was collected immediately after a standardized session of physiotherapy and was stored at ambient temperature and was processed within 4 h from collection. Fresh sputum (1 ml min) was mixed with an equal mass (1:1) of Sputasol (Oxoid SR089A, Oxoid Ltd., Poole, England) and was incubated in a water bath at 37°C for 15 min, before further qualitative processing for the detection of *Pseudomonas aeruginosa*. Processed sputa (10 μ l) were inoculated and incubated, onto several selective media for the isolation of *Pseudomonas aeruginosa*, including:- Columbia Blood Agar (Oxoid CM0331) supplemented with 5% (v/v) defribinated horse blood, MacConkey Agar (Oxoid CM0007) and *Pseudomonas *Isolation Agar (PIA) (Oxoid CM0559 + SR0102). All media were incubated aerobically at 37°C for 48 h, unless otherwise stated. The PIA plates were incubated at room temperature for a further three days following initial 48 hrs incubation. In addition, all different phenotypes from the sputum of each patient were identified phenotypically employing a combination of conventional identification methods (e.g. oxidase), as well as the API Identification schemes (API 20NE, API 20E) (Biomérieux, Les Halles, France).

### Molecular (PCR) detection of *Pseudomonas aeruginosa *from sputum

#### DNA extraction

All DNA isolation procedures were carried out in a Class II Biological Safety Cabinet (MicroFlow, England) in a room physically separated from that used to set up nucleic acid amplification reaction mixes and also from the "post-PCR" room in accordance with the Good Molecular Diagnostic Procedures (GMDP) guidelines of Millar *et al*[6], in order to minimise contamination and hence the possibility of false positive results. Bacterial genomic DNA was extracted directly from the patients' sputum, as well as from the reference strain *Pseudomonas aeruginosa *(Schroeter; Migula) ATCC 27853, by employment of the Roche High Purity PCR Template Preparation Kit (Roche, England), in accordance with the manufacturer's instructions. Extracted DNA was stored at -80°C prior to PCR amplification. For each batch of extractions, a negative extraction control containing all reagents minus sputum, was performed, as well as an extraction positive control with *P. aeruginosa*.

#### PCR amplification

All reaction mixes were set up in a PCR hood in a room separate from that used to extract DNA and the amplification and post-PCR room in order to minimise contamination. Initially PCR amplification conditions were optimised by separately varying magnesium chloride concentration, annealing temperature, primer concentration and DNA template concentration. Following optimisation, reaction mixes (100 μl) were set up as follows:-10 mM Tris-HCl, pH 8.3, 50 mM KCl, 2.5 mM MgCl_2_, 200 μM (each) dATP, dCTP, dGTP and dTTP; 1.25 U of *Taq *DNA polymerase (Amplitaq; Perkin Elmer), 0.1 μM (each) of the each set of primers (exoA &*opr*L) (Table 1) and 4 μl of DNA template. The reaction mixtures following a "hot start" were subjected to the following empirically optimized thermal cycling parameters in a Perkin Elmer 2400 thermocycler: 96°C for 5 min followed by 40 cycles of 96°C for 1 min, 55°C for 1 min, 72°C for 1 min, followed by a final extension at 72°C for 10 min. Positive (*P. aeruginosa *ATCC 27853 DNA) and multiple negative (water) amplification controls were included in every set of PCR reactions. In addition, a broad-range/universal PCR was employed with each sputum to demonstrate successful extraction of bacterial DNA from the specimen, as well as lack of PCR inhibition, by employing the highly conserved 16S rDNA primers, PSL/PSR (Table 1), as previously described [7]. Any sputum which failed to amplify this locus was re-extracted and amplified until a positive signal was obtained.

**Table 1:** Oligonucletoides and optimised experimental PCR amplification conditions

**Target**	**Gene locus**	**Primer 5'-----------------------3'**	**Optimum MgCl_2 _concentration (mM)**	**Annealing temperature**	**Size of Amplicon (bp)**
Eubacteria [7]	16S rRNA	f: 5'-AGG ATT AGA TAC CCT GGT AGT CCA-3'	2.5	55°C	312
		r: 5'-ACT TAA CCC AAC ATC TCA CGA CAC-3'			
*Pseudomonas aeruginosa *[8]	*opr*L^1^	f: 5'-ATG GAA ATG CTG AAA TTC GGC-3'	2.5	55°C	504
		r: 5'-CTT CTT CAG CTC GAC GCG ACG-3'			
*Pseudomonas aeruginosa *[9]	*exo*A^2^	f: 5'-GAC AAC GCC CTC AGC ATC ACC AGC-3'	2.5	72°C	396
		r: 5'-CGC TGG CCC ATT CGC TCC AGC GCT-3'			

^1^, outer membrane lipoprotein; ^2^, exotoxin A.

#### Detection of amplicons

Following amplification, aliquots (10 μl) were removed from each reaction mixture and examined by electrophoresis (80 V, 45 min) in gels composed of 2% (w/v) agarose (Gibco, UK) in TAE buffer (40 mM Tris, 20 mM acetic acid, 1 mM EDTA, pH 8.3), stained with ethidium bromide (5 μg/100 ml). Gels were visualised under UV illumination using a gel image analysis system (UVP Products, England) and all images archived as digital graphic files (*.bmp). Where a band was visualized at the correct expected size for either *exo*A or *opr*L loci, the specimen was considered positive for *P. aeruginosa*.

## Results and Discussion

A comparison of the occurrence of *P. aeruginosa *in patients' sputum by conventional and molecular techniques is shown (Table 2). By culture, there were 30 patients positive for *P. aeruginosa*, whereas by molecular techniques, there were 35 positive patients. In 39 patients (22 *P aeruginosa *[PA] positive [+ve] & 17 *P. aeruginosa *[PA] negative [-ve]), there was complete agreement between molecular and conventional detection techniques. The *opr*L locus was more sensitive than the ETA locus, as the former was positive in 10 more patients and there were no patients where the ETA was positive and the *opr*L target negative, However, there were five patients who were culture positive but PCR -ve by both gene targets. Given that the universal 16S rDNA PCR was positive for these sputum specimens and that the appropriate molecular controls were working optimally, this may accounted for by potential phenotypic misidentification of *P. aeruginosa*, which has been recently described.[10] Alternatively, discrepant results (PCR+ve/culture-ve) could reflect true *P. aeruginosa *colonization with a false-negative culture result due to sample overgrowth by other bacteria or to the presence of non-cultivable organisms or auxotrophic mutations in the organism. Where a PCR +ve/culture -ve result was recorded (10 patients), we followed these patients and recorded that five of these patients converted to being culture-positive at times ranging from 4–17 months later, with a mean lag time of 4.5 months, whereas the remaining five patients remained negative for *P. aeruginosa*.

**Table 2:** Comparison of conventional culture with molecular detection of *Pseudomonas aeruginosa *from the sputum of adult CF patients

**PCR (OprL)**	**PCR (exotoxin A)**	**Culture**	**Frequency of patients (%)**
+	+	+	22 (38.6%)
+	-	+	3 (5.3%)
-	-	+	5 (8.8%)
+	+	-	3 (5.3%)
+	-	-	7 (12.3%)
-	-	-	17 (29.7%)

In this study, two PCR assays were performed individually for the molecular detection of *P. aeruginosa *directly from the sputum of patients with CF. Two gene loci were targeted, as specific markers of *P. aeruginosa*, namely the exotoxin A (ETA) gene and the outer membrane lipoprotein (*opr*L) gene. ETA is produced by the majority of *P. aeruginosa *strains and can inhibit eucaryotic protein biosynthesis at the level of polypeptide chain elongation factor 2, similarly to diphtheria toxin.[9] OprL is an outer membrane lipoprotein which has been implicated in efflux transport systems, as well as affecting cell permeability.[8]

*Pseudomonas aeruginosa *is the most important bacterial pathogen in patients with CF [11], as demonstrated with high prevalence data in most of the National CF Registries. Chronic *Pseudomonas *colonisation of the major airways leading to delibating exacerbations of pulmonary infection, is the major cause of morbidity and mortality in patients with CF, hence it is important to be able to reliably detect *P. aeruginosa *from patients' sputum. Emerson *et al*. [12] recently published their findings of a US Cystic Fibrosis Foundation (CFF) registry-based study, which showed that infection related to *P. aeruginosa *was a major predictor of morbidity and mortality, whereby the 8-year risk of death parameter was 2.6 times higher in patients who had positive sputum cultures for this organism, as well as having a significantly lower percent predicted forced expiratory volume (FEV1). These workers suggested that early interventions may help decrease associated morbidity and mortality of young patients with CF.

More recently, Rosenfeld *et al*. [13] described the pathophysiology and risk factors for early *P. aeruginosa *infection in CF. These workers suggested that chronic lower airway infection with *P. aeruginosa *is associated with significant morbidity and mortality among CF patients [13]. However, they suggested that first acquisition of *P. aeruginosa *does not appear to cause an immediate and rapid decline in lung function, as early isolates are generally non-mucoid, antibiotic-sensitive and present at low densities, suggesting a possible "window of opportunity" for early intervention, as they describe [13]. This study also concluded that there are no controlled trials demonstrating clinical benefit in young children following early intervention, particularly for anti-pseudomonal therapy, as well as the long-term safety profile and optimal drug regimen.[13]

It is therefore important that primary diagnostic bacteriology laboratories have the ability to detect transient and early *Pseudomonas *colonization as early as possible, so that (i) aggressive antibiotic regimes may be considered,(ii). the patient is managed optimally, in an attempt to avoid early biofilm formation and chronic colonization with *Pseudomonas *and (iii) appr*opr*iate infection control precautions are considered. More recently, West *et al*. showed by a combination of serum IgG, IgA and IgM anti *P. aeruginosa *antibodies, in conjunction with these authors' Wisconsis Cystic Fibrosis Radiograph score, that *P. aeruginosa *infections occurred approximately 6–12 months before the organism was recovered from respiratory secretions.[4] In addition, this study demonstrated that mixing of young child with older chronically colonised children was associated with a significantly increased risk of *P. aeruginosa *acquisition.

Given that not all laboratories employ molecular detection methods for *Pseudomonas aeruginosa*, either from culture plates or patients' sputum, small numbers of *Pseudomonas *colonies (n = 1–2) may therefore be missed when present in the early stages of colonization preceding infection of the patient's airways, particularly where such single colonies are mixed alongside other phenotypically similar genera on the primary culture plate.[14] Pragmatic, practical and cost implications leave it impossible to qualitatively identify the total bacterial microflora present on non-selective primary plates from sputum. Therefore, any rapid molecular screening method should be encouraged to detect low copy numbers of organisms in the early stages of colonization/infection, where the main value of this diagnostic assay is in the rapid screening of patients with no or an intermittent history of *Pseudomonas *colonization. Although such assays are not generally available in most clinical diagnostic laboratories, these laboratories generally do have access to such technology at regional specialist microbiology centres and it may therefore be prudent to establish routine analysis of children's sputum, particularly from patients with no or an intermittent history by culture of *Pseudomonas *colonization, at annual review. Furthermore, it is very important to follow up any such patients, which demonstrate a PCR +ve/culture -ve finding from their sputum, in order to establish whether there is transient infection in patients, which never result in established colonization leading to chronic infection. Additionally, it is important to monitor such PCR +ve/culture -ve patients in terms of optimal antibiotic management and infection control.

Overall, our study demonstrated that molecular assays for the detection of *P. aeruginosa *were able to detect *P. aeruginosa *at an early stage, on average 4.5 months sooner than culture detection. However, it remains unclear what this "window of opportunity" potentially offers in terms of a decrease in *P. aeruginosa*-associated morbidity and mortality.

## Authors' contributions

JEM, PGM and JSE jointly conceived the study and designed the experiments. JX executed and analyzed all practical aspects of the study, as well as analyzing the data. JEM executed certain molecular components of experimentation, analyzed the data and prepared the manuscript. BCM helped guide the molecular aspects of experimentation and was involved in drafting of the manuscript. PGM provided expert microbiological analysis and interpretation of data. JSE provided clinical expertise, interacted with the CF patients and was involved in interpretation of data, as well as critically reviewing the final manuscript. All authors read and approved the final manuscript.
